# Deep learning model for predicting postoperative survival of patients with gastric cancer

**DOI:** 10.3389/fonc.2024.1329983

**Published:** 2024-04-02

**Authors:** Junjie Zeng, Dan Song, Kai Li, Fengyu Cao, Yongbin Zheng

**Affiliations:** Department of Gastrointestinal Surgery, Renmin Hospital of Wuhan University, Wuhan, Hubei, China

**Keywords:** deep learning, machine learning, gastric cancer, SEER, DeepSurv

## Abstract

**Background:**

Prognostic prediction for surgical treatment of gastric cancer remains valuable in clinical practice. This study aimed to develop survival models for postoperative gastric cancer patients.

**Methods:**

Eleven thousand seventy-five patients from the Surveillance, Epidemiology, and End Results (SEER) database were included, and 122 patients from the Chinese database were used for external validation. The training cohort was created to create three separate models, including Cox regression, RSF, and DeepSurv, using data from the SEER database split into training and test cohorts with a 7:3 ratio. Test cohort was used to evaluate model performance using c-index, Brier scores, calibration, and the area under the curve (AUC). The new risk stratification based on the best model will be compared with the AJCC stage on the test and Chinese cohorts using decision curve analysis (DCA), the net reclassification index (NRI), and integrated discrimination improvement (IDI).

**Results:**

It was discovered that the DeepSurv model predicted postoperative gastric cancer patients’ overall survival (OS) with a c-index of 0.787; the area under the curve reached 0.781, 0.798, 0.868 at 1-, 3- and 5- years, respectively; the Brier score was below 0.25 at different time points; showing an advantage over the Cox and RSF models. The results are also validated in the China cohort. The calibration plots demonstrated good agreement between the DeepSurv model’s forecast and actual results. The NRI values (test cohort: 0.399, 0.288, 0.267 for 1-, 3- and 5-year OS prediction; China cohort:0.399, 0.288 for 1- and 3-year OS prediction) and IDI (test cohort: 0.188, 0.169, 0.157 for 1-, 3- and 5-year OS prediction; China cohort: 0.189, 0.169 for 1- and 3-year OS prediction) indicated that the risk score stratification performed significantly better than the AJCC staging alone (P < 0.05). DCA showed that the risk score stratification was clinically useful and had better discriminative ability than the AJCC staging. Finally, an interactive native web-based prediction tool was constructed for the survival prediction of patients with postoperative gastric cancer.

**Conclusion:**

In this study, a high-performance prediction model for the postoperative prognosis of gastric cancer was developed using DeepSurv, which offers essential benefits for risk stratification and prognosis prediction for each patient.

## 1 Introduction

With an estimated 10,000+ new cases annually, gastric cancer is the sixth most common malignancy diagnosed worldwide (1). Because gastric cancer is often advanced at diagnosis, the mortality rate is high, making it the third most common cause of cancer-related death. Age-standardized 5-year net survival rates for gastric cancer generally range from 20-40%, with significant variation in Asia (2). Surgery is the sole option for treatment; however, even after a total resection, recurrence is frequent (3, 4). Although the surgery level has improved recently, the overall prognosis is poor. Also, gastric cancer’s intra-tumour, intra-patient, and inter-patient heterogeneity poses a severe obstacle to developing targeted drugs (3, 5, 6). Gastric cancer remains a significant burden on society, and there is a need to improve the treatment of this disease (7).

In this context, a feasible postoperative prognostic model for gastric cancer may be beneficial for the clinical management of gastric cancer. Zhang et al. (8) developed a nomogram based on Cox risk regression to assess the postoperative prognosis of patients with early gastric cancer with a C-index of 0.730. Liu et al. (9) developed a nomogram to predict patients with early gastric cancer after surgery based on data from a multi-center study and achieved satisfactory results. Most of the clinical prediction models developed so far for the prognosis of patients with postoperative gastric cancer are based on Cox risk regression models in nomograms. This algorithm may limit the performance of the prediction models.

Artificial neural networks, a subset of machine learning, process signals in individual neurons and link them to parameterize the weights of input data, enabling identification of highly complex linear and nonlinear relationships (10). Deep learning networks can identify intricate correlations between mortality risk and predictive clinical variables, even providing specific suggestions based on assessed application risk (11). Additionally, Katzman et al. (12) created the Deep Learning Survival Neural Network (DeepSurv), a novel deep learning approach incorporating Cox proportional risk for survival analysis. The authors demonstrate that DeepSurv performs on par with or better than existing survival models and may be used to prescribe treatments for better survival results. There are no reports on using Deepsurv for postoperative stomach cancer prognosis.

This study intends to provide a postoperative predictive model for stomach cancer based on deep learning algorithms using data from the Surveillance, Epidemiology, and End Results (SEER) program database. This study also developed a prediction tool based on the DeepSurv algorithm to provide physicians and patients with individualized survival prediction information.

## 2 Materials and methods

### 2.1 Study population

**Figure 1:**
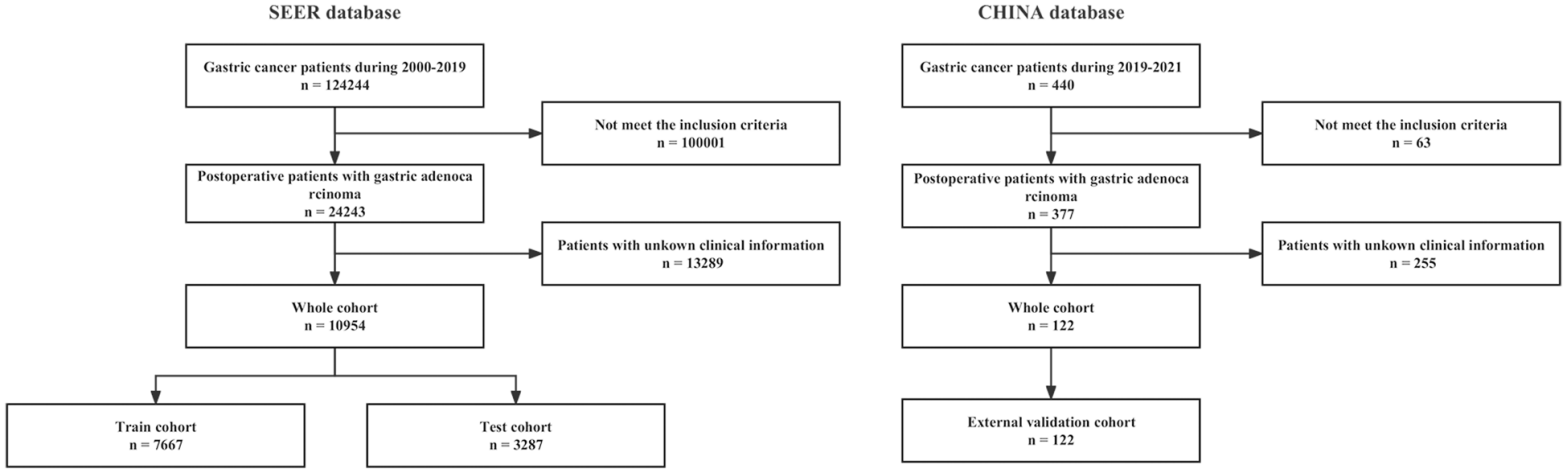
The flowchart of data filtering.

The SEER public database is representative of the US population, and patient data were obtained from multi-center population data such as rural and urban. Postoperative prognosis of gastric Cancer cases and their details were retrieved between 2000 and 2019 using SEER*Stat version 8.4.1 software. Clinical cases were included if the following criteria were met: (1) site and morphological code “stomach”; (2) histological codes including 8140/3, 8141/3, 8142/3, 8143/3, 8144/3, 8262/3, and 8323/3 [International Classification of Diseases for Oncology, Third Revision (ICD-O-3)] (3) Pathologically confirmed malignant tumor with first primary tumor. (4) Age greater than 20 years. Exclusion criteria were: (1) missing demographic information, such as gender, marital status, and race; (2) Information on the cause of death or subsequent survival was unavailable; (3) patients who lack clinical knowledge, such as histopathological information, surgical information, tumor primary site code, T stage (AJCC stage 7), N stage (AJCC stage 7) or clinical grade. The China database included patients diagnosed with gastric cancer and treated surgically at the People’s Hospital in Hubei Province from November 2009 to May 2021 and was completely different from the SEER database. Data filtering for the Chinese database also followed the above inclusion-exclusion criteria. All patients gave verbal informed consent before data collection and after approval from the Institutional Review Board of the People’s Hospital of Hubei Province. The study’s endpoint was OS (overall survival), the time interval from first diagnosis to death. This study followed the Strengthening the Reporting of Observational Studies in Epidemiology (STROBE) reporting guidelines. The data screening process is exhibited in **Figure 1**.

### 2.2 Variables

Demographic data, such as age, race, gender, marital status, and clinical features, such as AJCC stage (AJCC stage 7), T stage, N stage, M stage, tumor location, tumor size, and clinical grade, were gathered. Additionally, characteristics related to surgery were maintained for the number of surgically inspected lymph nodes and the frequency of positive lymph nodes. The research also included records on radiation and chemotherapy.

### 2.3 The development of models

In a 7:3 ratio, the cases from the SEER database were randomly split into training and test cohorts. Three different prediction models were constructed in this study, namely, the Cox risk regression model based on the linear prediction model (13), the Random Survival Forest (RSF) model based on the machine learning algorithm, and the DeepSurv model based on a deep learning algorithm (14–17). The three prediction models are based on the algorithm’s characteristics to select suitable variables for the best prediction performance. Cox regression model variable selection is based on single-factor and multi-factor regression analysis. In contrast, the RSF model uses lattice search in combination with K-cross validation to select the best combination of variables. The DeepSurv model is built on a neural network approach, where all the variables may be incorporated into the training without being chosen first. No selection is required. Meanwhile, grid search optimizes RSF and DeepSurv models with hyperparameters. Model training and hyperparameter tuning are done on the training set.

### 2.4 The evaluation and interpretation of models

The model’s effectiveness was assessed in the test cohort and the China cohort. The consistency index (C-index) and area under the operating characteristic curve (1, 3, and 5 years) were utilized as assessment measures (18, 19). Brier scores and calibration curves were used to assess the model’s calibration. The closer the score is to zero, the more accurate the model (20). Permutation Importance plots show the weights of the variables involved in the modeling to interpret the model (21).

### 2.5 The DeepSurv risk stratification of patients

The DeepSurv risk score, which measures the postoperative damage to patients, is based on the projected number of events at a particular endpoint node in the DeepSurv model. Using X-tile software (22) using their risk ratings, we classified the patients in the high-risk, medium-risk, and low-risk. The DeepSurv risk stratification was examined using the log-rank test and the Kaplan-Meier curve survival analysis.

The clinical advantages and usefulness of the risk stratification in comparison to AJCC tumor staging alone were assessed using the net reclassification index (NRI), integrated discrimination improvement (IDI), and decision curve analysis (DCA). NRI and IDI are substitutes for AUC that may be used to gauge how well a new model predicts risks and how beneficial it is (23, 24). DCA is a technique for estimating net benefits at various threshold probabilities and assessing the therapeutic value of alternative models (25, 26). The curves for the treat-none plan (representing no clinical benefit) and the treat-all-patients scheme (showing the highest clinical expenses) were presented as examples.

### 2.6 The individual prediction

The DeepSurv model was used in this work to construct an interactive prediction tool that provides survival predictions for specific patients. There are two components to this interface: (1) a user information input interface and (2) an interface for presenting survival prediction results. The information input interface is designed to guide the input of information on clinical variables relevant to the modeling. The results display interface provides individualized survival predictions based on the patient information entered after the user clicks the prediction button. All SEER-related codes follow the SEER guidelines.

### 2.7 Statistical analysis

The Wilcoxon test was used to compare differences in demographic and clinical data between the training and validation sets, and the 2 test or Fisher’s exact test was used to compare differences in categorical data. Statistical significance was defined as a two-tailed p-value less than 0.05. The models were produced using Python (version 3.7). Based on the pysurvival modules (version 0.17.2), the Cox, RSF, and DeepSurv models. The first data analysis used r (version 4.2.3) and SPSS—data visualization based on GraphPad Prism 9. The interactive prediction tool relied on Streamlit (https://streamlit.io/) for its construction.

## 3 Results

### 3.1 The characteristics of patients

A total of 11,076 patients with postoperative gastric cancer were included in the study. The study comprised 10,954 patients in total using data from the SEER database. The primary clinical baseline features of the patients are shown in **Table 1**. Most of the patients were white (7151[64.56%]). Overall, male patients were the majority (7449 [67.25%]). Most patients did not receive radiation therapy (7458 [67.33%]). One hundred twenty-two patients with postoperative gastric cancer were included in the Chinese database, and most patients had a tumor pathological grade III/IV (87 [71.31%]). Almost all patients did not receive radiotherapy (113 [96.62%]).

**Table 1:** The information for post-operative gastric cancer patients in the training cohort, the test cohort and China cohort.

Characteristics		Train cohort	Test cohort	China cohort
		n = 7667	n = 3287	n = 122
Age				
	20-69	3962(51.68%)	1727(52.54%)	74(60.66%)
	70+	3705(48.32%)	1560(47.46%)	48(39.34%)
Sex				
	Female	2574(33.57%)	1011(30.76%)	42(34.43%)
	male	5093(66.43%)	2276(69.24%)	80(65.57%)
Marital status				
	Married	4840(63.13%)	2137(65.01%)	114(93.44%)
	Unmarried	2827(36.87%)	1150(34.99%)	8(6.56%)
Race				
	American Indian/Alaska Native	57(0.74%)	19(0.58%)	0
	Asian or Pacific Islander	1661(21.66%)	679(20.66%)	122(100%)
	Black	992(12.94%)	395(12.02%)	0
	White	4957(64.65%)	2194(66.75%)	0
Primary Site				
	Cardia/Fundus	2592(33.81%)	1108(33.71%)	20(16.39%)
	Body/antrum/pylorus/Lesser/Greater	4173(54.43%)	1758(53.48%)	99(81.15%)
	Overlapping/Unspecified	902(11.76%)	421(12.81%)	3(2.46%)
Grade				
	I/II	3349(43.68%)	1483(45.12%)	35(28.69%)
	III/IV	4318(56.32%)	1804(54.88%)	87(71.31%)
Summary stage				
	Distant	906(11.87%)	342(10.40%)	0
	Localized	2371(30.92%)	988(30.06%)	38(31.15%)
	Regional	4390(57.26%)	1957(59.54%)	84(68.85%)
T stage				
	T1	1640(21.39%)	714(21.72%)	29(23.77%)
	T2	2503(32.65%)	1059(32.22%)	13(10.66%)
	T3	2432(31.72%)	1034(31.46%)	25(20.49%)
	T4	1092(14.24%)	480(14.60%)	55(45.08%)
N stage				
	N0	2986(38.95%)	1254(38.15%)	39(31.97%)
	N1	2521(32.88%)	1106(33.65%)	23(18.85%)
	N2	1267(16.53%)	558(16.98%)	20(16.39%)
	N3	893(11.65%)	369(11.23%)	40(32.79%)
M stage				
	M0	6919(90.24%)	3007(91.48%)	122(100%)
	M1	784(10.23%)	280(8.52%)	0
AJCC stage				
	I	2339(30.51%)	1002(30.48%)	28(22.95%)
	II	2012(26.24%)	869(26.44%)	23(18.85%)
	III	2260(29.48%)	1004(30.54%)	71(58.20%)
	IV	1056(13.77%)	412(12.53%)	0
Radiation				
	No	5143(67.08%)	2202(66.99%)	113(96.62%)
	Yes	2524(32.92%)	1085(33.01%)	9(7.38%)
Chemotherapy				
	No	3868(50.45%)	1664(50.62%)	60(49.18%)
	Yes	3799(49.55%)	1623(49.38%)	62(50.82%)
Tumor size				
	< 5 cm	4452(58.07%)	1931(58.75%)	67(54.92%)
	5 cm +	3215(41.93%)	1356(41.25%)	55(45.08%)
Regional nodes examined				
	<30	4517(58.91%)	1965(59.78%)	26(21.31%)
	30 +	3150(41.09%)	1322(40.22%)	96(78.69%)
Lymph node metastasis rate				
	0	3274(42.70%)	1393(42.38%)	42(34.43%)
	<30	2149(28.03%)	927(28.20%)	41(33.61%)
	30-70	1417(18.48%)	607(18.47%)	27(22.13%)
	>70	827(10.79%)	360(10.95%)	12(9.84%)

### 3.2 The development of models

Deepsurv model: After grid search and hyperparameter search for optimization, the skeleton part of the deep learning model has four layers with nodes 41, 84, 23, and 49 from top to bottom. It constitutes a fully linked feedforward neural network that undertakes the prognostic prediction task (**Supplementary Figure S1**). Cox model: Age, gender, marital status, race, pathological tumor grade, TNM stage, AJCC stage, radiation, chemotherapy, tumor size, number of lymph nodes examined, and lymph node positivity rate were all significant factors according to univariate Cox regression analysis (**Supplementary Table S1**). In multivariate Cox regression analysis, age, chemotherapy, AJCC stage, T, M, AJCC stage, radiotherapy, chemotherapy, lymph nodes evaluated in number, and positive lymph node rate were identified as prognostic factors after gastric cancer surgery (**Supplementary Table S2**). RSF model: Using grid search and K-fold validation, a total of 16 variables were selected for the RSF model, including age, gender, marital status, race, primary tumor site, tumor pathological grade, summary stage, TNM stage, AJCC stage, radiotherapy, chemotherapy, tumor size, number of lymph nodes examined and lymph node positivity rate (**Supplementary Table S3**).

### 3.3 The evaluation and interpretation of the models

**Figure 2:**
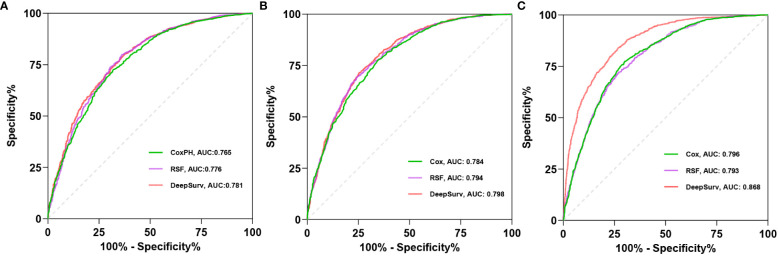
The receiver operating curves (ROC) for 1- **(A)**, 3- **(B)**, 5-year **(C)** OS survival predictions for three models in test cohort.

**Figure 3:**
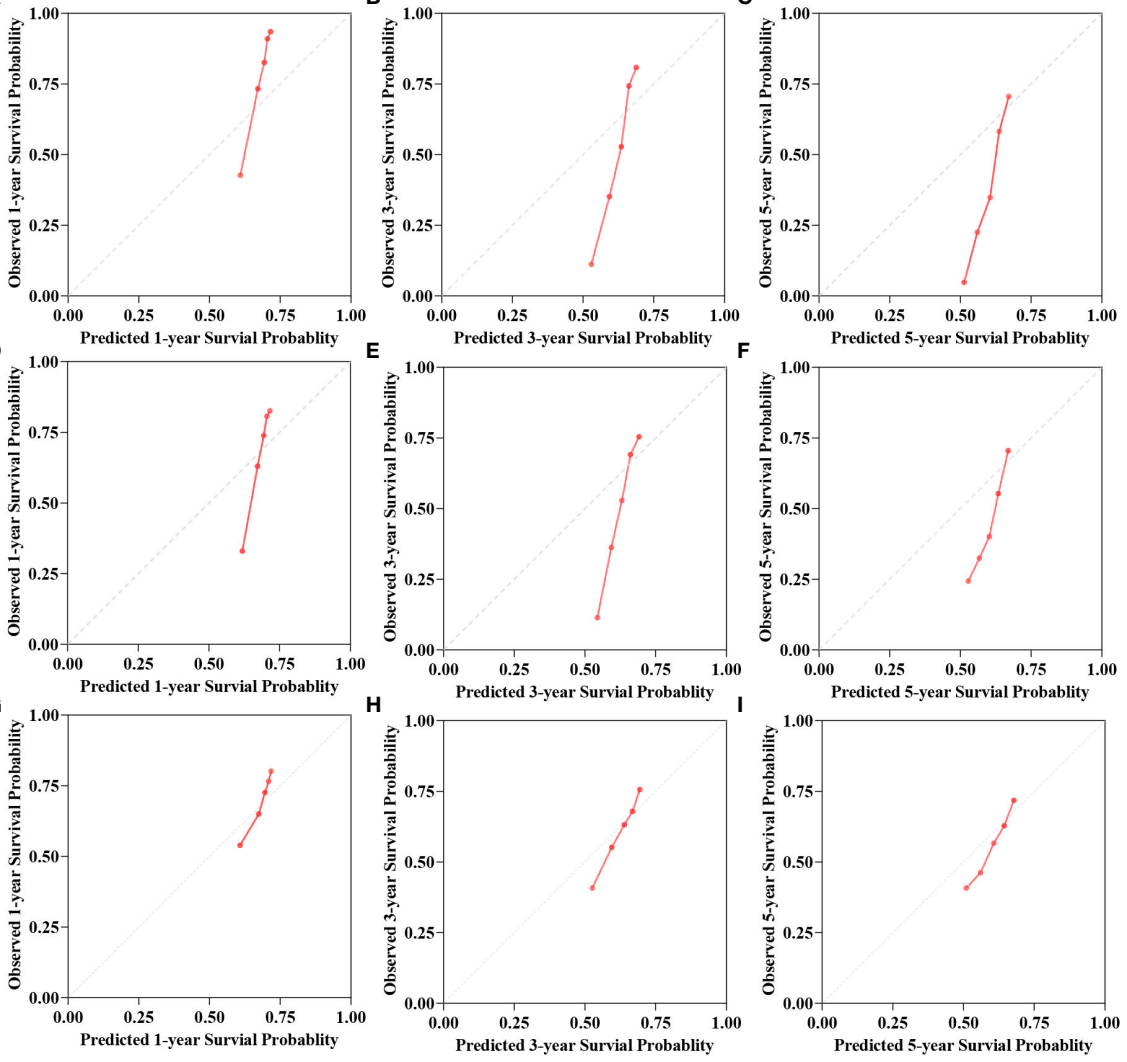
The calibration curves for 1-, 3-, 5-year OS survival predictions for three models in test cohort. **(A–C)** is the calibration curves for Cox model. **(D–F)** is the calibration curves for RSF model. **(G–I)** is the calibration curves for DeepSurv model.

C-index, AUC, and Brier scores are used to compare the predictive performance of the models. The results demonstrate that the Deepsurv model exhibits advantages over the Cox and RSF models (**Table 2**). The C-index of the Deepsurv model in the test cohort is 0.787. The 1-year, 3-year, and 5-year OS prediction AUCs are 0.781, 0.798, and 0.868, respectively (**Figure 2**). The Brier score of the model is less than 0.25, which indicates that the model is well-calibrated. The brier score of the DeepSuv model reached 0.149, 0.189, and 0.183 in predicting 1-, 3-, and 5-year OS, respectively, which was the smallest among the three models. The calibration curves showed that in the validation cohort, the DeepSurv model predicted and observed survival probabilities with better agreement than the other models (**Figure 3**). These findings were confirmed in the China cohort (**Table 2**), demonstrating that the DeepSurv model performed and calibrated better than the Cox and RSF models (**Supplementary Figures S2**, **S3**).

**Table 2:** The models’ Brier score and C-index in the Test cohort and China cohort.

Index		Test cohort		DeepSurv model	China cohort		DeepSurv model
		Cox model	RSF model		Cox model	RSF model	
Brier Score							
	For 1‐year OS	0.179	0.169	0.149	0.147	0.132	0.117
	For 3‐year OS	0.221	0.219	0.189	0.224	0.207	0.188
	For 5‐year OS	0.216	0.213	0.183			
C‐index		0.746	0.755	0.787	0.786	0.791	0.821

**Figure 4:**
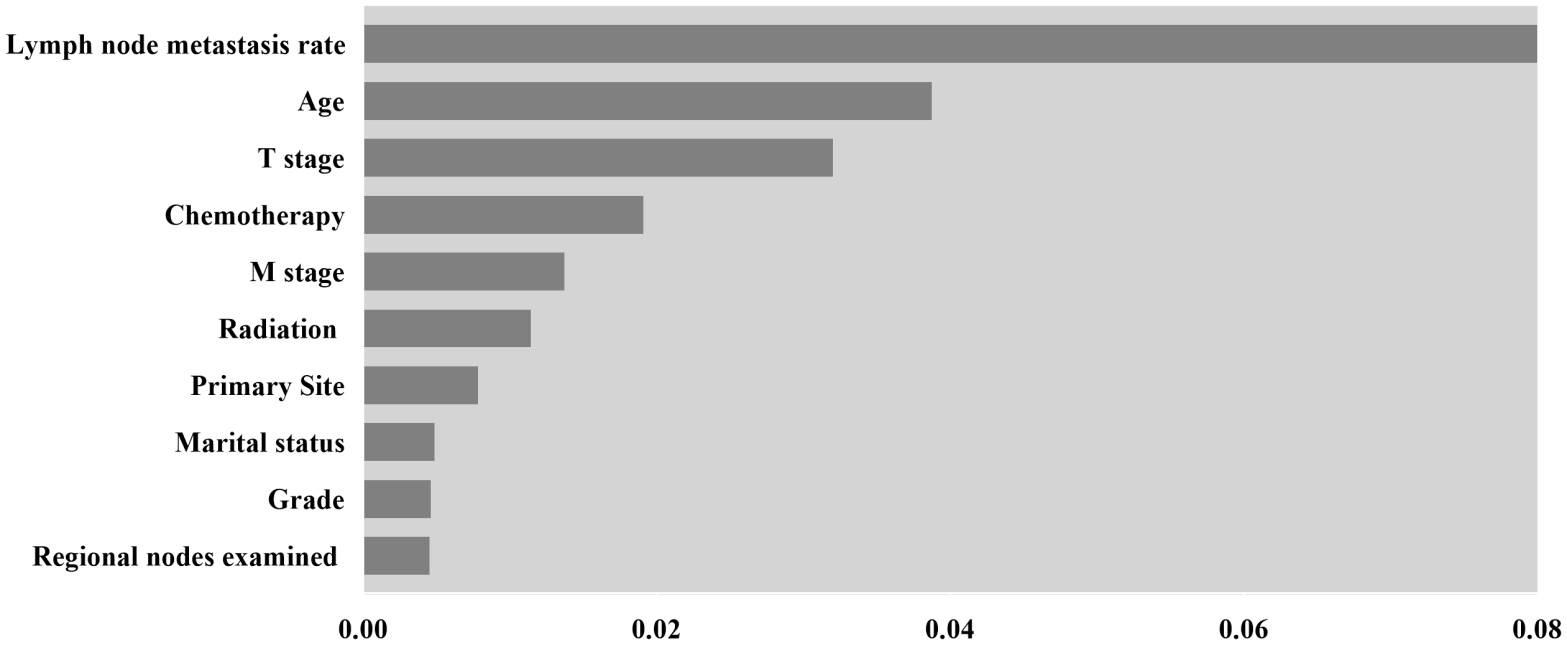
Feature importance for DeepSurv model, only the top 10 variables in importance are shown in the Figure.

In addition, this study mapped the importance of model features to interpret the DeepSurv model. The top ten variables in descending order of importance are presented in the feature importance plot (**Figure 4**). Before age, T-stage, and chemotherapy, the lymph node positive rate was deemed to have the most significant impact on the model’s ability to predict outcomes.

### 3.4 The Deepsurv risk stratification of patients

**Figure 5:**
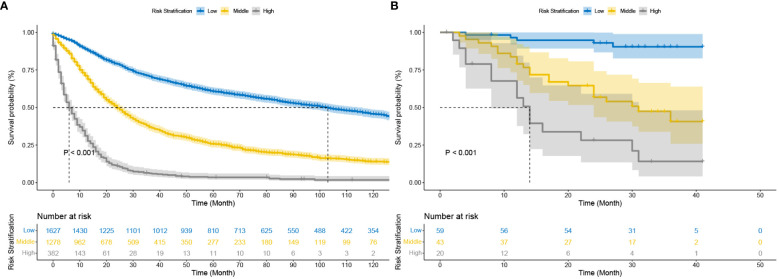
Kaplan–Meier curves of cancer-specific survival for new risk classification **(A)** The DeepSurv risk stratification stage in the test cohort; **(B)** The DeepSurv risk stratification in the China cohort.

Stratification of patients is essential to guide patient management. The X-tile technique was used to categorize patients into three groups: high-risk (score >2.3), medium-risk (score 0.6 to 2.3), and low-risk (score <0.6) (**Supplementary Figure S4**). More information about the X-tile is described in additional material. **Figure 5** shows the outcomes of the Kaplan-Meier survival analysis and log-rank test for the high-risk, intermediate-risk, and low-risk groups. The Kaplan‐Meier OS curves showed significant discrimination among the three risk groups in both the test and China cohorts.

The C-index, NRI, and IDI changes were used to compare the accuracy between the risk stratification and the AJCC staging alone. While using the risk stratification in the test cohort, the C-index was 0.777, the NRI for the 1-, 3- and 5-year OS were 0.399 (95% CI = 0.340‐0.463), 0.288 (95% CI = 0.230‐0.337) and 0.267 (95% CI = 0.216‐0.312), and the IDI values for 1-, 3- and 5-year OS were 0.188 (95% CI = 0.170‐0.209, P < 0.05),0.169 (95% CI = 0.150‐0.188, P < 0.05) and 0.157 (95% CI = 0.134‐0.180, P < 0.05) (**Table 3**). These findings, confirmed in the China cohort (**Table 3**), showed that risk stratification was more accurate in predicting prognosis than AJCC staging.

**Table 3:** C‐index, NRI, and IDI of the DeepSurv risk stratification and AJCC stage in survival prediction for postoperative prognosis of gastric cancer.

Index		Testing cohort			P value	China cohort			P value
		Estimate	95% CI			Estimate	95% CI		
NRI (vs. the AJCC tumor staging)									
	For 1‐year OS	0.399	0.340‐0.463			0.399	0.335‐0.459		
	For 3‐year OS	0.288	0.230‐0.337			0.288	0.236‐0.335		
	For 5‐year OS	0.267	0.216‐0.312						
IDI (vs. the AJCC tumor staging)									
	For 1‐year OS	0.188	0.170‐0.209		**<0.05**	0.189	0.086‐0.305		**<0.05**
	For 3‐year OS	0.169	0.150‐0.188		**<0.05**	0.169	0.050‐0.300		**<0.05**
	For 5‐year OS	0.157	0.134‐0.180		**<0.05**				
C‐index									
	The risk stratification	0.777				0.791			
	The AJCC stage	0.652				0.677			

**Figure 6:**
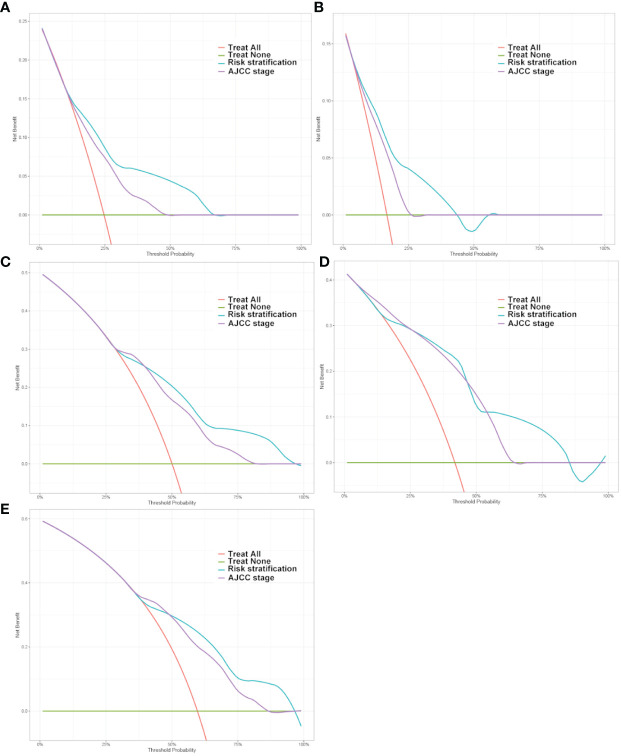
Decision curve analysis of the DeepSurv risk stratification and AJCC tumor staging for the survival prediction of Postoperative patients with gastric cancer. **(A, C, E)** 1‐year, 3‐year and 5‐year survival benefit in the test cohort. **(B, D)** 1‐year and 3‐year survival benefit in the China cohort.

The clinical benefits of the risk stratification were compared with those of the AJCC stage. DCA curves showed that the risk stratification could better predict the 1-, 3- and 5-year OS, as it added more net benefits compared with the AJCC stage for almost all threshold probabilities in both the test and China cohorts, and with both the treat-none and the treat-all patients schemes (**Figure 6**).

### 3.5 The individual postoperative prognostic prediction

**Figure 7:**
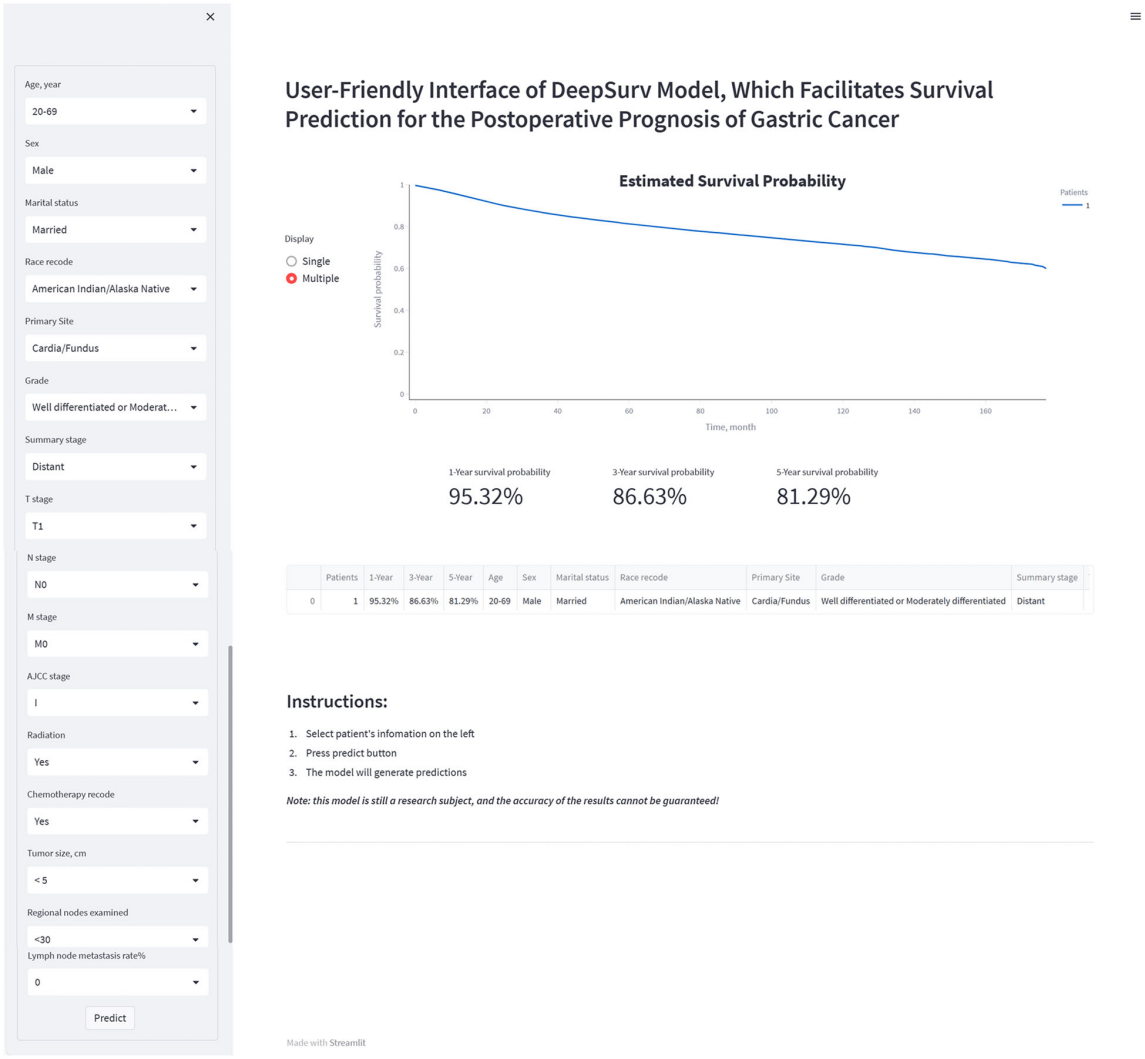
The manual interactive interface based on DeepSurv model for predicting the survival probabilities of postoperative patients with gastric cancer.

The study developed a manual interactive interface based on the trained Deepsurv model for predicting the probability of survival of patients with gastric cancer after surgery (**Figure 7**). The analysis’s findings are represented graphically as a survival curve, which displays patient survival rates at 1, 3, and 5 years after surgery below the graph and shows the likelihood of survival with time for patient inputs. The ability to fit different patient survival curves into the same chart is also provided to facilitate patient comparison. (Github: https://github.com/DrZJJ/GC_SURG).

## 4 Discussion

This study reports on the SEER database’s DeepSurv algorithm-based prognostic model for postoperative gastric cancer. Compared with Cox regression and RSF models, the DeepSurv model shows advantages in predicting the overall survival of patients with gastric cancer after surgery. The lymph node positivity rate was determined to be the most critical risk factor, with age, T-stage, and the total number of positive lymph nodes coming in second and third. The DeepSurv model-based risk classification and individual postoperative prognostic survival prediction showed promise for clinical use.

Deep learning network models might become increasingly popular as a fresh analytical technique to assist clinical judgment (10, 27–29). The effectiveness of deep learning models in enhancing treatment results is a crucial problem that requires consistently being evaluated in practice (12, 30, 31). Following is a summary of the benefits of deep learning network models for postoperative prognosis prediction in surgical research. First, real-world clinical factors and other non-linearly associated variables can be accommodated by DeepSurv. In contrast to previous models, deep learning algorithms may incorporate nonlinear risk functions related to outcomes. Second, DeepSurv is adaptable in how it handles challenging clinical circumstances. The DeepSurv model can assess censored variables and automatically learn feature representations from clinical data that has not been interpreted. Additionally, in-depth data analysis has demonstrated that DeepSurv model predictions perform better. The benefits of DeepSurv models in managing important factors and sample sizes may be crucial in biological marker research due to their capacity to learn factor representations.

The DeepSurv prediction model’s risk factor analysis may aid surgical management and lessen the load on the medical system. The lymph node positivity rate had a decisive impact on the predictive outcome of the model according to the importance ranking of the mapped model features. The length of time that patients survived was dramatically shortened as the rate of lymph node-positive rose. Additionally, previous research has suggested that the proportion of individuals with stomach cancer who have positive lymph nodes may independently predict their prognosis and risk of recurrence (32–35). However, the impact of specific lymph node positivity rates on patient prognosis still needs further study.

Whether or not patients have received chemotherapy after gastric cancer surgery also significantly impacts the prognosis prediction of patients, according to the model’s special importance demonstration. The benefit of neoadjuvant and adjuvant chemotherapy in treating gastric cancer has been shown (36, 37). However, further discussion is needed regarding the optimal timing of chemotherapy, the benefit of radiotherapy, the minimum required range of lymph node dissection, and the optimal chemotherapy regimen. Due to the limitations of the seer database, we did not obtain more detailed information about the patient’s chemotherapy (38, 39). Whether the patient received neoadjuvant chemotherapy, the chemotherapy dose, and the chemotherapy cycle are information that will help us further refine the prognostic prediction model.

Deepsurv risk stratification allows the prognosis of postoperative patients to be assessed based on baseline information and information related to surgical pathology. The DeepSurv risk stratification approach offers advancements in the field of algorithms in addition to being a more typical risk stratification method based on columnar plots. However, it is challenging to accurately assess each scoring system’s effectiveness because of the variability of the factors included. There is still a need to compare the characteristics of each scoring system in a large population. Doctors can evaluate their patient’s chances of survival by utilizing the DeepSurv risk stratification. Additionally, given the median survival time for patients in high-risk groups (risk scores greater than 2.3) is just eight months, doctors should pay closer attention to these patients. Early death is more likely for them. The patient’s postoperative prognosis may be given from a more precise viewpoint by having the network prediction tool generate individual survival probability curves. The postoperative prognosis prediction alone offers a more particular picture of the patient’s prognosis.

Our model demonstrated advantages compared to recent related studies that predicted postoperative survival of gastric cancer patients. Wang utilized a column-line graph approach to construct a predictive model for postoperative survival in patients with gastric cardia adenocarcinoma, achieving a maximum c-index of 0.746 (40). Similar results were obtained in the study by Nie et al (41). And in this study, the best C-index of DeepSurv model reached 0.821. Liu et al., on the other hand, employed a machine learning algorithm to develop a prognostic model for postoperative gastric cancer with an AUC value of up to 0.8; however, their evaluation was solely based on the AUC value (42). Models were evaluated comprehensively, including C-index, AUC, IDI, NRI and DCA. It is important to note that objective factors such as different selected datasets and included variables may impact the performance of models. Therefore, further comprehensive discussion is warranted when comparing it with other similar studies.

Some limitations of the present study should be mentioned. First, due to the study’s retrospective design, there may be a selection bias. Second, the fact that the training and test sets were taken from the same database may have lowered the model’s generalizability. The data for the external validation set were also from the same Chinese hospital, and the sample size needs to be further expanded. Third, some possible variables, such as drug use and genetic factors, were not available due to the limitations of the SEER database. The DeepSurv model may perform better when more possible variables are included. Another flaw in our study is that adjuvant treatments (both adjuvant and neoadjuvant) were not further investigated.

## Data availability statement

The original contributions presented in the study are included in the article/**Supplementary Material**. Further inquiries can be directed to the corresponding author.

## Ethics statement

The studies involving humans were approved by The Institutional Review Board of the People’s Hospital of Hubei Province (Apptoval No. WDRY2023-K145). The studies were conducted in accordance with the local legislation and institutional requirements. Written informed consent for participation was not required from the participants or the participants’ legal guardians/next of kin in accordance with the national legislation and institutional requirements.

## Author contributions

JZ: Writing – review & editing. DS: Writing – review & editing. KL: Writing – review & editing. FC: Writing – review & editing. YZ: Writing – review & editing, Writing – original draft.
