# Supplementary material for: Deep learning model for predicting postoperative survival of patients with gastric cancer
Source: Front Oncol. 2024 Apr 2;14:1329983. doi: 10.3389/fonc.2024.1329983 (PMC11018873; doi:10.3389/fonc.2024.1329983)
Supplement: Supplementary file 1 [file DataSheet_1.docx]

**Deep learning Model for Predicting Postoperative Survival of Patients with Gastric Cancer**

**Junjie Zeng, MD** ^1^**, Kai Li, MD** ^1^**, Fengyu Cao, MD** ^1^**, Yongbin Zheng, MD, PhD** ^1,^*****

^1^ Department of Gastrointestinal Surgery, Renmin Hospital of Wuhan University, Wuhan, Hubei, China.

* Corresponding authors: Yongbin Zheng, MD, PhD, Department of Gastrointestinal Surgery, Renmin Hospital of Wuhan University, Wuhan, Hubei, 430060, China. Email: [yongbinzheng@whu.edu.cn](mailto:yongbinzheng@whu.edu.cn)

**Table S1** The results of the univariable Cox regression analysis

| **Variable** | **HR** | **95% CI** | ***P* value** |
| --- | --- | --- | --- |
| Age | 1.53 | 1.45 - 1.62 | <0.001 |
| Gender | 1.08 | 1.02 - 1.05 | 0.010 |
| Marital status | 1.24 | 1.18 -1.32 | <0.001 |
| Race recode | 1.11 | 1.07 - 1.15 | <0.001 |
| Grade | 1.46 | 1.39 - 1.55 | <0.001 |
| T stage | 1.47 | 1.44 - 1.52 | <0.001 |
| N stage | 1.53 | 1.49 - 1.57 | <0.001 |
| M stage | 2.95 | 2.72 - 3.21 | <0.001 |
| AJCC stage | 1.63 | 1.60 - 1.69 | <0.001 |
| Radiation | 0.87 | 0.83 - 0.93 | <0.001 |
| Chemotherapy | 0.94 | 0.89 - 1.00 | 0.041 |
| Tumor size | 1.49 | 1.41 -1.58 | <0.001 |
| Regional nodes examined | 0.99 | 0.99 - 1.00 | <0.001 |
| Lymph node metastasis rate | 1.72 | 1.68 - 1.78 | <0.001 |

**Table S2** The results of the multivariable Cox regression analysis

| **Variable** | **HR** | **95% CI** | ***P* value** |
| --- | --- | --- | --- |
| Age | 1.61 | 1.52 - 1.71 | <0.001 |
| Gender | 1.30 | 1.22 - 1.38 | <0.001 |
| Marital status | 1.24 | 1.17 - 1.32 | <0.001 |
| Race recode | 1.13 | 1.09 - 1.17 | <0.001 |
| Grade | 1.20 | 1.13 - 1.27 | <0.001 |
| T stage | 1.21 | 1.16 - 1.26 | <0.001 |
| N stage | 1.01 | 0.95 - 1.07 | 0.714 |
| M stage | 1.38 | 1.23 - 1.55 | <0.001 |
| AJCC stage | 1.18 | 1.12 - 1.26 | <0.001 |
| Radiation | 0.89 | 0.83 - 0.96 | 0.003 |
| Chemotherapy | 0.72 | 0.67 - 0.78 | <0.001 |
| Tumor size | 1.01 | 0.95 - 1.08 | 0.6753 |
| Regional nodes examined | 0.98 | 0.98 - 0.99 | <0.001 |
| Lymph node metastasis rate | 1.32 | 1.26 - 1.39 | <0.001 |

**Table S3** RSF model variable selection results.The RSF model searches for the best combination of constituent model variables based on grid search and K-fold cross-validation. The best results were obtained for the 18th time (K = 17) when the composition of the variables was age, gender, marital status, race, primary tumor site, tumor pathological grade, summary stage, TNM stage, AJCC stage, radiotherapy, chemotherapy, tumor size, number of lymph nodes examined and lymph node positivity rate..

| **K** | **Mean fit time** | | **Mean score time** | **Mean test score** |
| --- | --- | --- | --- | --- |
| 0 | 17.338859 | | 0.147638 | 0.720407 |
| 1 | 17.299353 | | 0.158735 | 0.799659 |
| 2 | 17.500834 | | 0.155572 | 0.814604 |
| 3 | 19.613478 | | 0.176112 | 0.795952 |
| 4 | 19.266633 | | 0.181676 | 0.797071 |
| 5 | | 19.150619 | 0.191137 | 0.800256 |
| 6 | | 19.283414 | 0.184448 | 0.803429 |
| 7 | | 18.239021 | 0.174029 | 0.80431 |
| 8 | | 18.993842 | 0.190284 | 0.805819 |
| 9 | | 19.190534 | 0.191637 | 0.807191 |
| 10 | | 22.19435 | 0.164064 | 0.809818 |
| 11 | | 22.122755 | 0.18026 | 0.809908 |
| 12 | | 21.279265 | 0.162609 | 0.81118 |
| 13 | | 21.352123 | 0.163804 | 0.811006 |
| 14 | | 21.076367 | 0.179235 | 0.812141 |
| 15 | | 21.186235 | 0.163964 | 0.809944 |
| 16 | | 21.16561 | 0.166022 | 0.812686 |
| 17 | | 21.023466 | 0.167279 | 0.816526 |


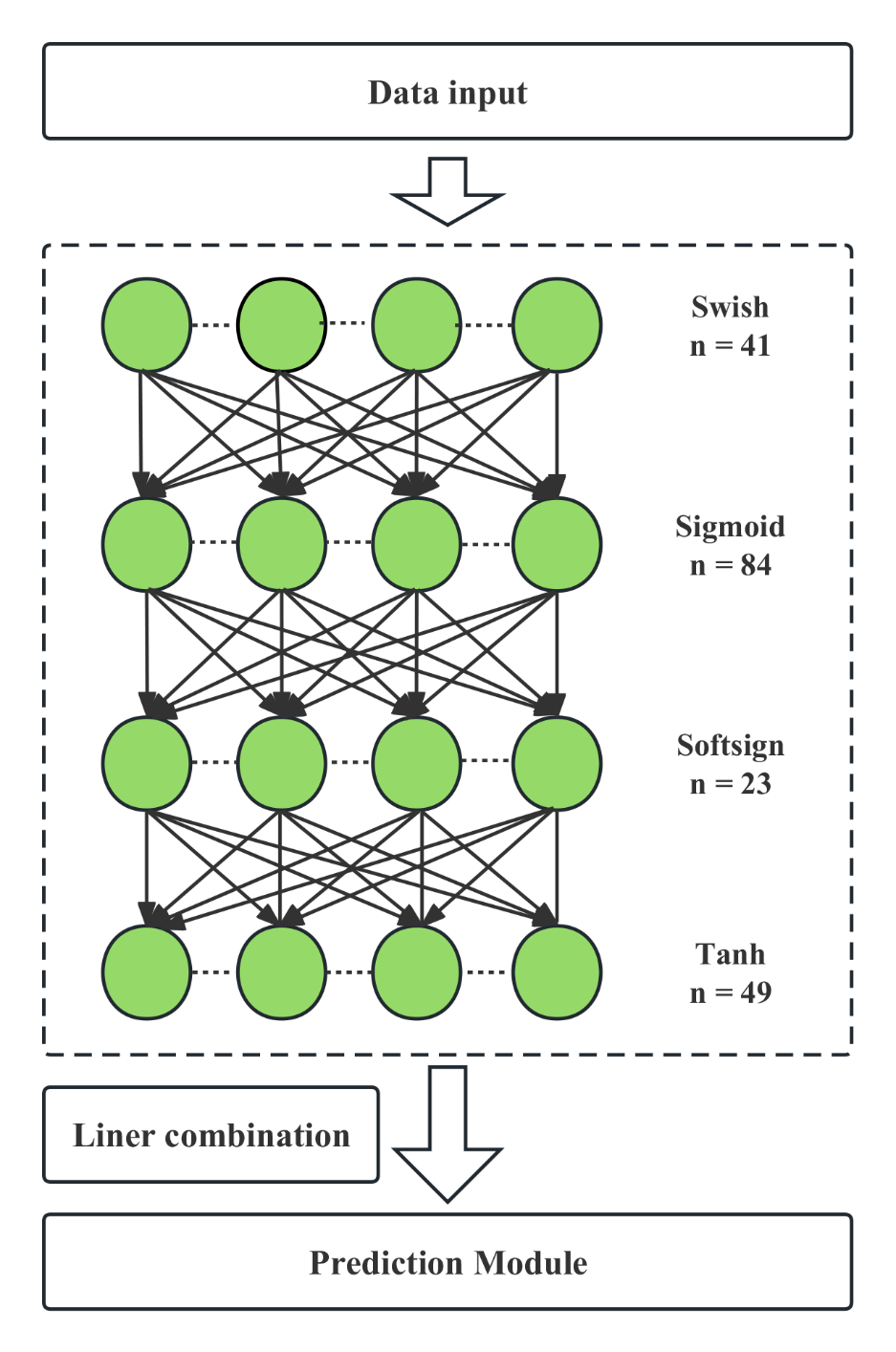


**Figure S1** Diagram of the deep learning procedure. The model hidden layer is illustrated in the dashed box. The type of activation and the number of nodes used in each layer after optimization are labeled on the right.


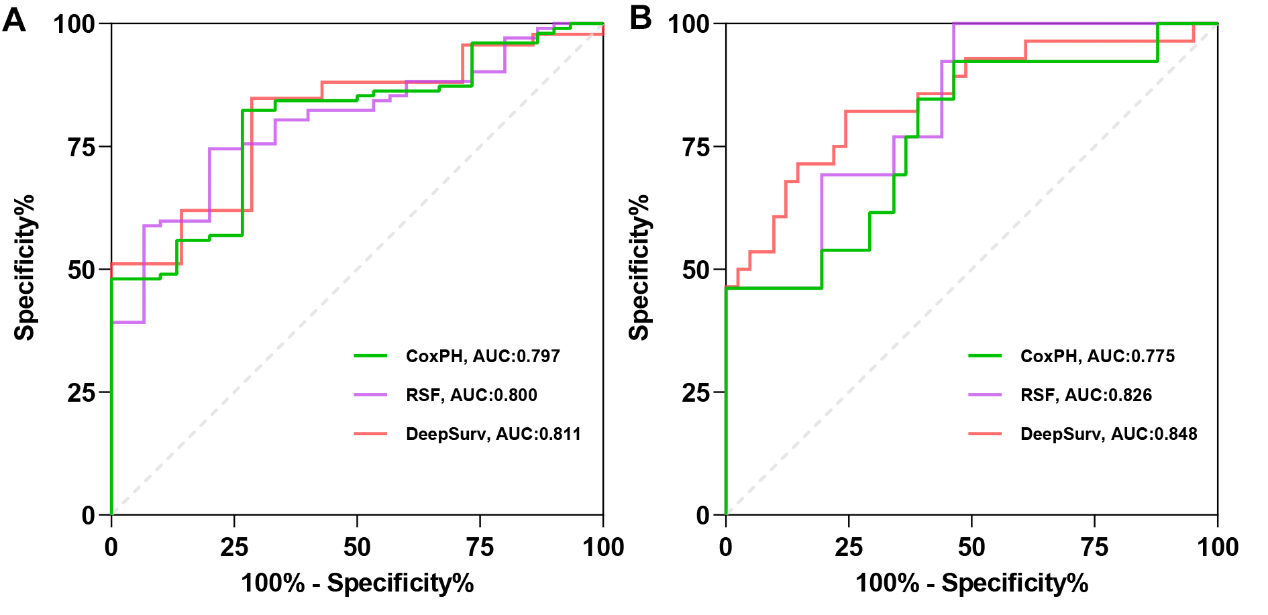


**Figure S2** The receiver operating curves (ROC) for 1- (A), 3-year (B) OS survival predictions of three models in China cohort.


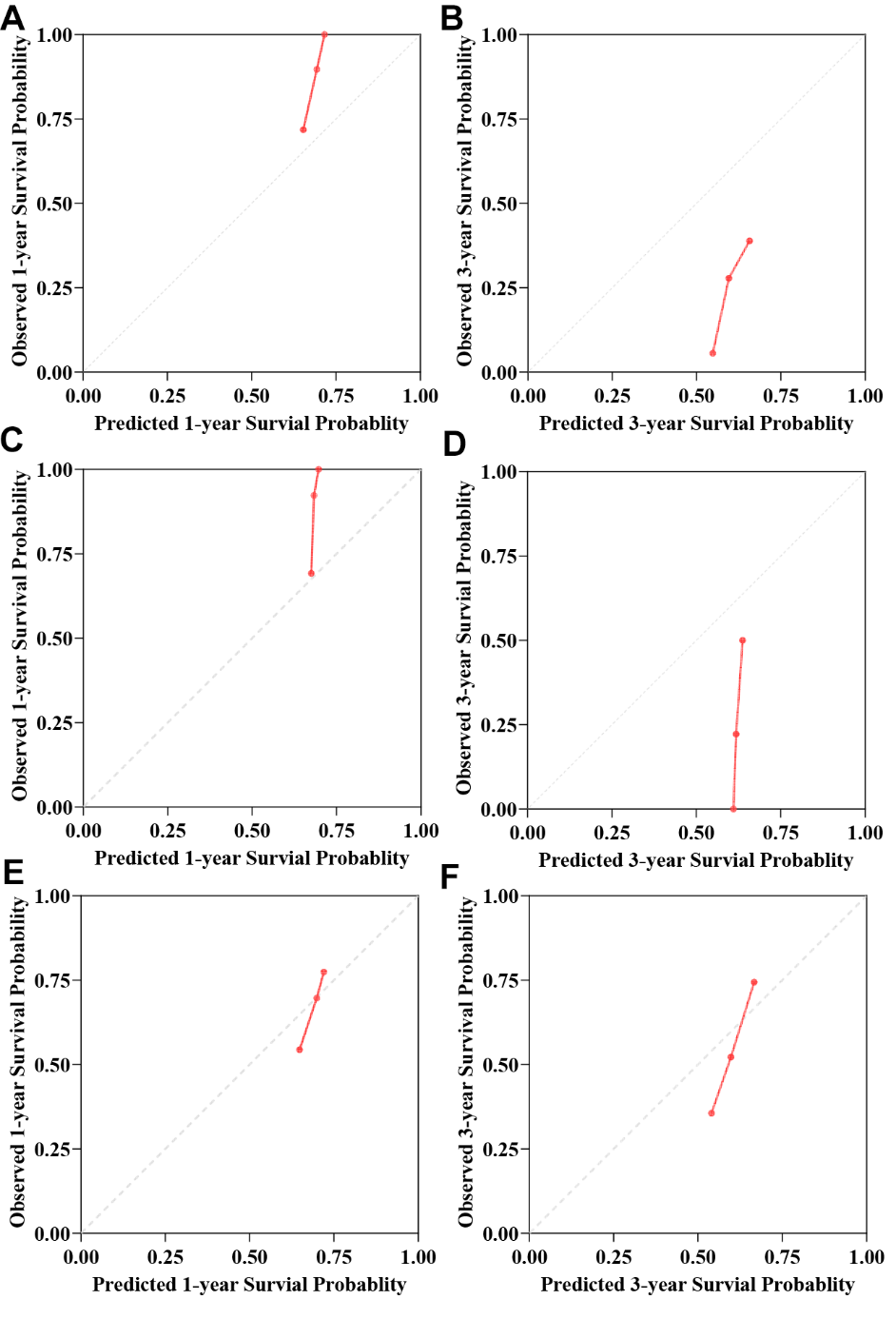


**Figure S3** The calibration curves for 1-, 3- year OS survival predictions of three models in China cohort. (A), (B) is the calibration curves for Cox model. (C), (D) is the calibration curves for RSF model. (E), (F) is the calibration curves for DeepSurv model.


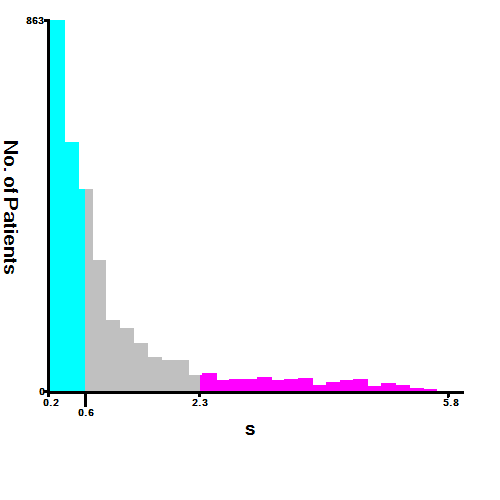


**Figure S4** The basis for grouping DeepSurv Risk Stratification (Cut-off point selected using X-tile).
